# Complete mitochondrial genome of *Turritella terebra bacillum*

**DOI:** 10.1080/23802359.2016.1144088

**Published:** 2016-06-20

**Authors:** Lin Zeng, Yonghong Wang, Jianshe Zhang, Changwen Wu

**Affiliations:** National Engineering Research Center of Marine Facilities Aquaculture, Zhejiang Ocean University, Zhoushan, Zhejiang, People’s Republic of China

**Keywords:** Mitochondrial genome, *Turritella terebra bacillum*, phylogenetic tree

## Abstract

The complete mitochondrial genome of the *Turritella terebra bacillum* was 15 868 bp in length and contained 13 protein-coding genes, 22 transfer RNA genes and two ribosomal RNA genes. The overall base composition of *T. terebra bacillum* was A 28.85%, T 35.88%, C 16.21% and G 19.06%. Phylogenetic tree construction indicated that *T. terebra bacillum* was most closely related to *Volutharpa perryi*. This molecular information will contribute to better understand its evolution and population genetics.

*Turritella terebra bacillum* (Kiener 1844), which belongs to Prosobranchia, Mesogastropoda, Turritellidae, has certain value of nourishment and pharmaceutical. It is a fairly widespread species in southeast coastal region in China (Ma & Feng 1993; Xie et al. 1996). In order to obtain genetic information and understand the evolution of *T. terebra bacillum*, we sequenced its complete mitochondrial genome (GenBank accession no. KU221394).

*Turritella terebra bacillum* were obtained in Mazhan town, Wenzhou city, Zhejiang province (27°10'31.39''N, 120°32'22.61''E) and were stored in the fish herbarium of Zhejiang Ocean University. Initially, the spiral shell was identified based on both the morphologic features and the *COX1* mitochondrial gene. Tissue samples for molecular analysis were reserved in absolute ethyl alcohol. The complete mitochondrial genome of *T. terebra bacillum* was extracted using the phenol–chloroform method. The PCR products were sequenced by Sanger’s method.

The complete mitochondrial genome of *T. terebra bacillum* was 15 868 bp in length, containing 13 protein-coding genes, 22 transfer RNA genes (tRNA) and two ribosomal RNA genes (rRNA). The mitogenome base composition was A 28.85%, T 35.88%, C 16.21% and G 19.06%, A + T content (64.73%) was remarkably higher than the G + C content (36.53%), which was similar to other mollusks (Boore 2006; Jiang et al. 2015). Thirteen protein-coding genes can be classified into two categories: *COX1, COX2, ND4L, ND4, ND5, ND2, ATP8, ATP6* and *ND3* were encoded by the light-strand, *COX3, CYTB, ND6* and *ND1* were encoded by the heavy strand. Eight protein-coding genes (*COX1, COX2, ND4L, ND5, ND2, ATP8, ATP6* and *ND3*) started with an ATG initiation codon, four protein-coding genes (*COX3, CYTB, ND6* and *ND1*) started with a CAT initiation codon, and only ND4 used GTG as the initiation codon. Seven protein-coding genes (*COX1, COX2, ND4L, ND4, ND2, ATP6* and *ND3*) used TAA as the termination codon, *ND5* and *ATP8* used TAG as the termination codon, and four genes (*COX3, CYTB, ND6* and *ND1*) used TTA as the termination codon. The two ribosomal RNA genes, 16SrRNA (1357 bp) was located between *tRNA^Leu^* and *tRNA^Val^* genes and 12SrRNA (949 bp) was located between *tRNA^Thr^* and *tRNA^Ser^* genes.

In the mitochondrial phylogeny, *T. terebra bacillum* was most closely related to *Volutharpa perryi*. *Thais clavigera* was placed at the most basal position forming an individual clade, while other species formed another large cluster (Figure 1).

**Figure 1.**
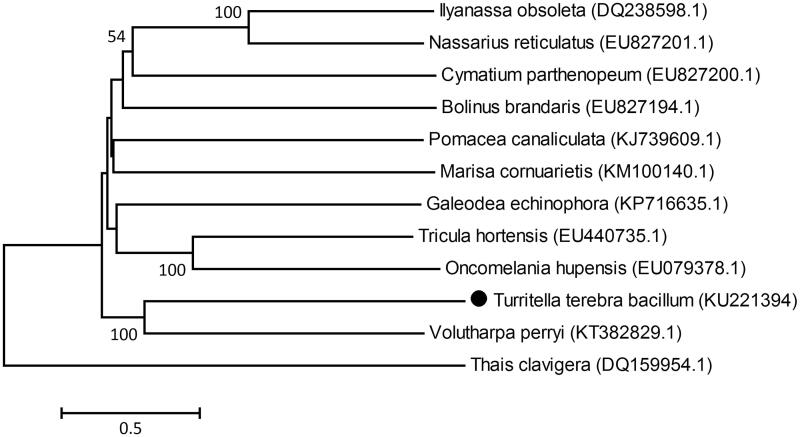
Phylogenetic tree of *Turritella terebra bacillum* was constructed with the Neighbor-Joining method using the program MEGA 4.0. The numbers at each branch indicate the percentage bootstrap values.
